# Supplementary material for: In Silico Analysis Predicts a Limited Impact of SARS-CoV-2 Variants on CD8 T Cell Recognition
Source: Front Immunol. 2022 Apr 27;13:891524. doi: 10.3389/fimmu.2022.891524 (PMC9094405; doi:10.3389/fimmu.2022.891524)
Supplement: Supplementary file 1 [file DataSheet_1.docx]

Supplementary Material


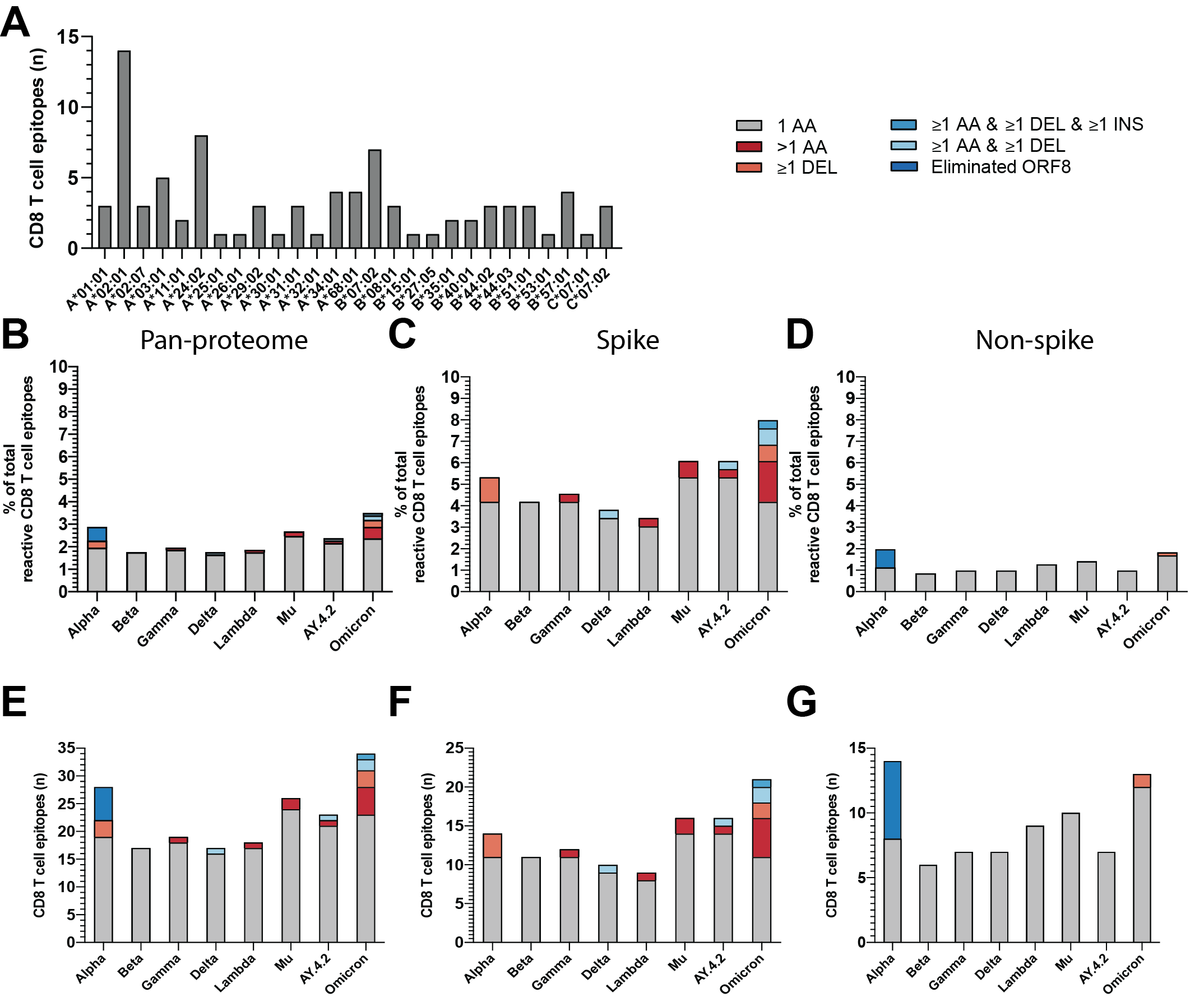


**Supplementary Figure 1.** **CD8 T cell recognized epitopes that overlap with a mutation**

**(A)** Numbers of unique CD8 T cell recognized epitopes included in this study that bind the indicated HLA restriction elements, based on literature. **(B)** Percentage of pan-proteome, spike **(C)**, non-spike **(D)** CD8 T cell recognized epitopes per variant that harbor the indicated types of mutations. Total epitope numbers are 973, 263 and 710, respectively. **(E)** Numbers of pan-proteome, **(F)** spike, **(G)** non-spike CD8 T cell recognized epitopes per variant that harbor the indicated categories of mutations.


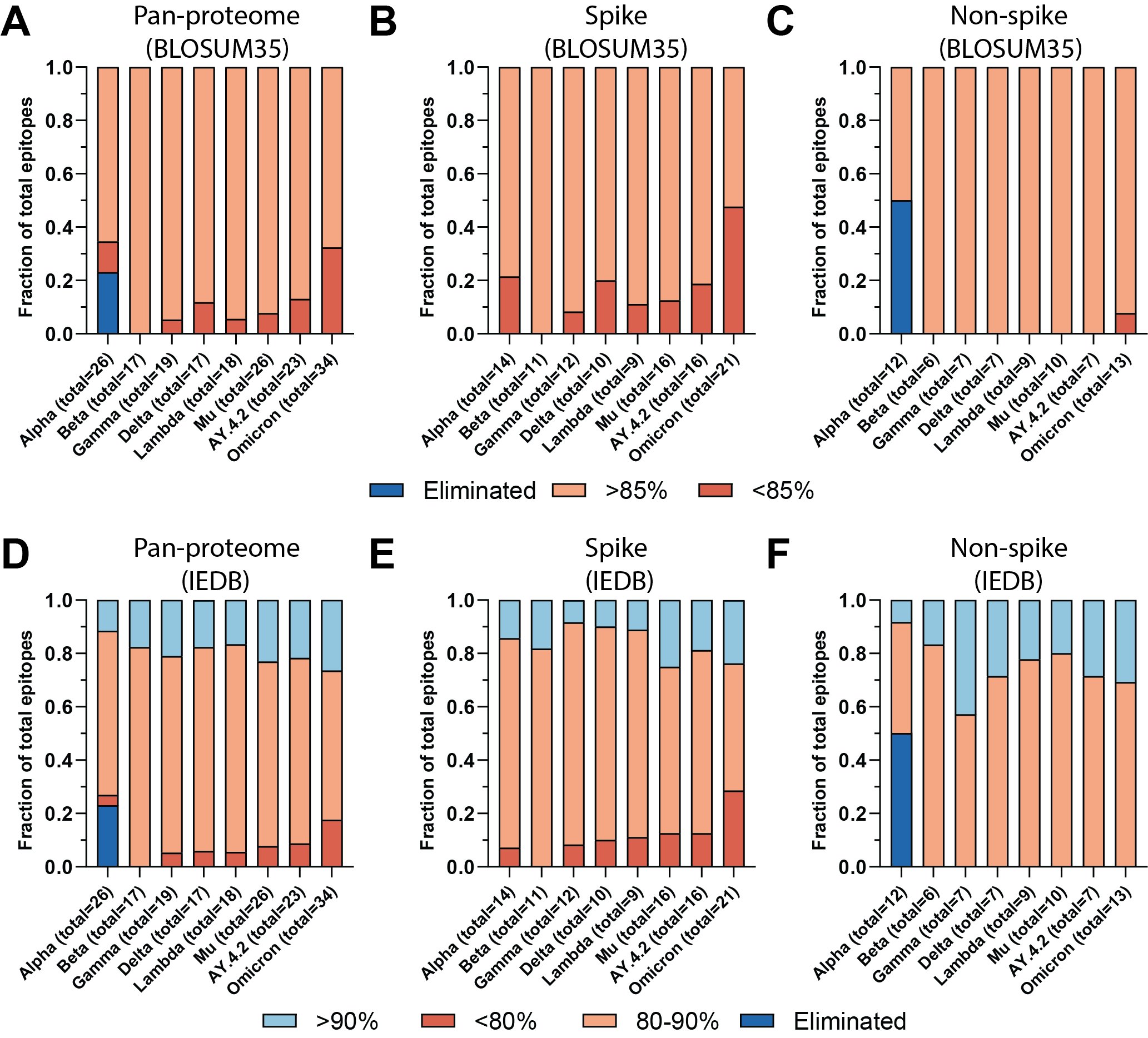


**Supplementary Figure 2.** **Mutant epitope sequence similarity to reference epitope sequence.**

**(A)** Fractions of pan-proteome, spike **(B)**, non-spike **(C)** CD8 T cell recognized epitopes where the sequence similarity of the altered epitope to the reference epitope was below or above 85%, using the method by Frankild et al. described in the text. **(D)** Fractions of pan-proteome, spike **(E)**, non-spike **(F)** CD8 T cell recognized epitopes where the sequence similarity of the altered epitope to the reference epitope was below 80%, above 90% or between 80-90%, using the IEDB epitope clustering tool described in the text. Epitopes were considered eliminated as a result of the ORF8 Q27* stop codon mutation (Alpha, n = 6).


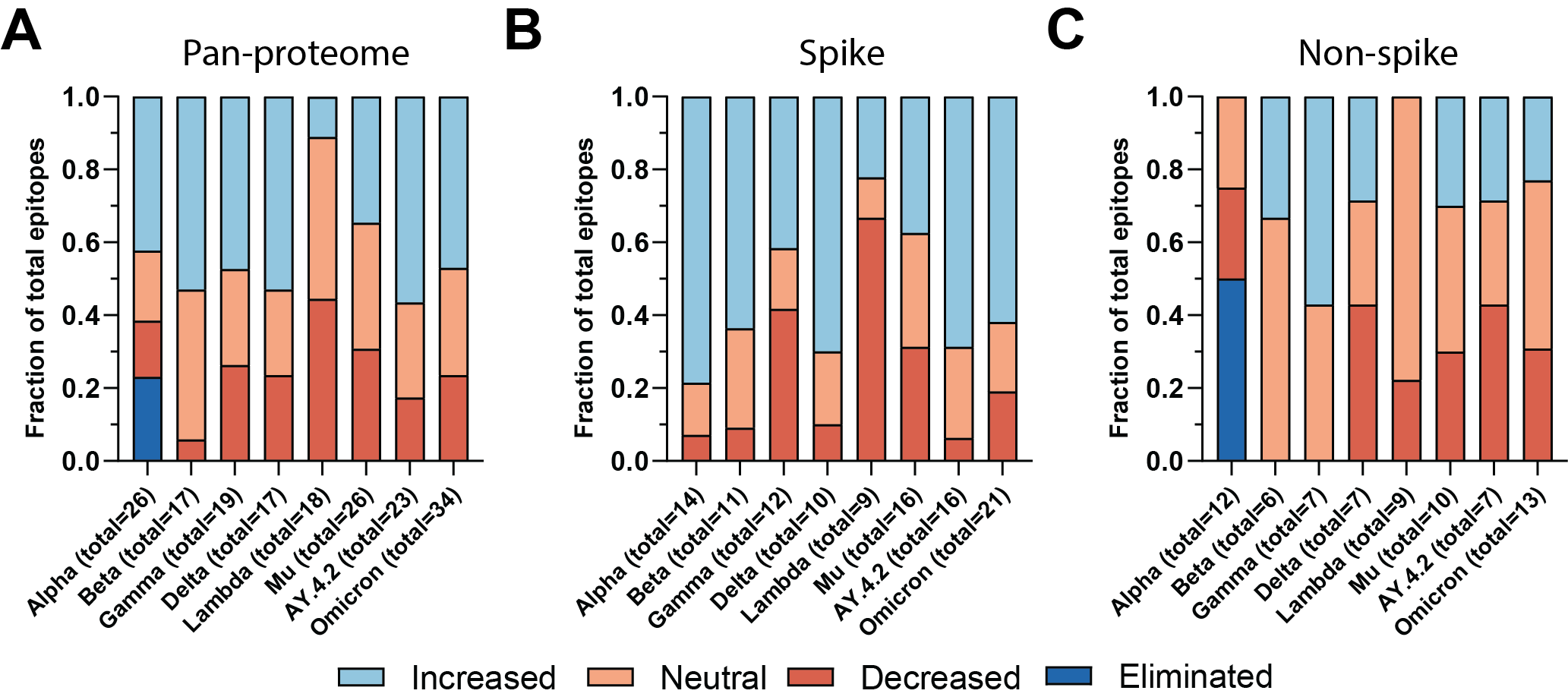


**Supplementary Figure 3.** **Fraction of altered CD8 T cell recognized epitopes with a change in predicted immunogenicity**

**(A)** Fractions of pan-proteome, spike **(B)**, non-spike **(C)** CD8 T cell recognized epitopes where the predicted immunogenicity increased, decreased or was unchanged as a result of the overlapping mutation.

|  | **SARS-CoV-2 variant** | | | | | | | |
| --- | --- | --- | --- | --- | --- | --- | --- | --- |
|  | **Alpha** | **Beta** | **Gamma** | **Delta** | **Lambda** | **Mu** | **AY.4.2** | **Omicron** |
| **ORF1ab** | T1001I, A1708D, I2230T, DEL3675/3677, P4715L | T265I, K1655N, K3353R, DEL3675/3677, P4715L | S1188L, K1795Q, DEL3675/3677, *P4715L*, E5665D | A1306S, P2046L, P2287S, V2930L, T3255I, T3646A, P4715L, G5063S, P5401L, A6319V | T1246I, P2287S, F2387V, L3201P, T3255I, G3278S, DEL3675/3677, P4715L | T1055A, T1538I, T3255I, Q3729R, P4715L, P5743S | A1306S, P2046L, P2287S, A2529V, V2930L, T3255I, T3646A, P4715L, G5063S, P5401L, A6319V | K856R, S2083I, DEL2084/2084, A2710T, T3255I, P3395H, DEL3674/3676, I3758V, P4715L, I5967V |
| **S** | DEL69/70, DEL144/144, N501Y, A570D, *D614G*, *P681H*, *T716I*, *S982A*, D1118H | D80A, D215G, K417N, E484K, N501Y, D614G, A701V | L18F, T20N, P26S, D138Y, R190S, K417T, E484K, N501Y, D614G, H655Y, T1027I, V1176F | T19R, E156G, DEL157/158, L452R, T478K, D614G, P681R, D950N | G75V, T76I, R246N, DEL247/253, L452Q, F490S, D614G, T859N | T95I, Y144S, Y145N, R346K, E484K, N501Y, D614G, P681H, D950N | T19R, T95I, G142D, Y145H, E156G, DEL157/158, A222V, L452R, T478K, D614G, P681R, D950N | A67V, DEL69/70, T95I, G142D, DEL143/145, N211I, DEL212/212, INS214EPE, G339D, S371L, S373P, S375F, S477N, T478K, Q493R, G496S, Q498R, N501Y, Y505H, T547K, D614G, H655Y, N679K, P681H, D796Y, N856K, Q954H, N969K, L981F |
| **ORF3a** |  | Q57H, S171L | S253P | S26L |  | Q57H, DEL256/257 | S26L |  |
| **E** |  | P71L |  |  |  |  |  | T9I |
| **M** |  |  |  | I82T |  |  | I82T | D3G, Q19E, A63T |
| **ORF7a** |  |  |  | V82A, T120I |  |  | V82A, T120I |  |
| **ORF7b** |  |  |  | T40I |  |  | T40I |  |
| **ORF8** | Q27*, R52I, Y73C, S84L | S84L | S84L, E92K | S84L, DEL119/120 | S84L | T11K, P38S, S67F, S84L | S84L, DEL119/120 | S84L |
| **N** | D3L, R203K, G204R, S235F | T205I | P80R, R203K, G204R | D63G, R203M, G215C, D377Y | P13L, R203K, G204R, G214C | T205I | D63G, R203M, G215C, D377Y | P13L, DEL31/33, R203K, G204R |

**Supplementary Table 1.** **SARS-CoV-2 variant amino acid changes**

*Highlighted in blue and underlined: mutations overlapping with CD8 T cell epitopes*

** stop codon*
